# Impact of Away Rotations on Emergency Medicine Match Rates in the Virtual Residency Recruitment Era

**DOI:** 10.5811/westjem.62267

**Published:** 2026-10-18

**Authors:** Jennifer Kaminsky, Stephen Thomas, Leslie Bilello, Nicole M. Dubosh

**Affiliations:** Beth Israel Deaconess Medical Center/Harvard Medical School, Department of Emergency Medicine, Boston, Massachusetts

## Abstract

**Introduction:**

Since the COVID-19 pandemic, many emergency medicine (EM) residencies have transitioned to virtual interviews, providing less in-person exposure for applicants. Away rotations offer applicants the opportunity to gain in-person exposure to residency programs beyond the virtual interview environment. Whether the away rotation is more important now and leads to higher rotator match rates in the virtual recruitment era remains unclear and warrants investigation. Our objective was to determine the percentage of EM interns who completed an in-person rotation at the residency program in which they matched and compare this to pre-COVID-19 match rates.

**Methods:**

This was a multicenter pilot study of EM residency programs. We recruited a convenience sample of academic programs from the eight Society for Academic Emergency Medicine (SAEM) US geographic regions. Inclusion criteria included academic EM programs that conducted virtual-only interviews post-COVID-19. We collected data via a survey form for the 2017–2024 application years regarding the number of matched interns who completed an in-person rotation at the program and the number of matched interns who were “home” versus “away” rotators. The first three years were defined as pre-COVID and the last three as post-COVID. The 2020–2021 academic year was excluded given the elimination of most away rotations during this cycle. We compared proportions of matched interns before and after COVID-19.

**Results:**

Eleven of 13 invited programs (representing seven of eight SAEM regions) participated in our analysis. Pre-COVID, 95 of 384 interns (24.7%; 95% CI, 20.5–29.4%) completed a rotation at their respective residency program. Post-COVID, this decreased by 2.86% (absolute risk difference; 95% CI, −6.13% to 4.02%; McNemar’s *P* = .06) to 84 of 384 (21.9%; 95% CI, 17.8–26.3%). Focusing solely on “away” rotators, 48 of the matched interns (12.5%; 95% CI, 9.4–16.2%) completed a rotation at their residency program pre-COVID compared to 47 interns (12.2%; 95% CI, 9.1–15.9%) post-COVID (*P* = .82). The absolute risk difference for the away rotators was 0.7% (95% CI, −10.6% to 9.1%, *P* = .82).

**Conclusion:**

In this pilot study, we found no statistically significant difference in the proportion of matched interns who completed an in-person rotation in the pre- versus post-COVID-19 era.

## INTRODUCTION

Before the onset of the COVID-19 pandemic, residency interviews were nearly universally held in-person. This allowed applicants to visit prospective programs and interact with program leadership, faculty, and current residents face-to-face. However, the pandemic prompted a shift in this traditional in-person format, posing an unprecedented and unique set of challenges for both applicants and training programs across all specialties.^1^,^2^ Previous studies have found that interview day environment, including format, structure, and on-site experiences have an impact on applicants’ ranking of programs.^3^–^5^

Organizations and working groups including the Association of American Medical Colleges, Council of Residency Directors in Emergency Medicine, Clerkship Directors in Emergency Medicine, and Coalition for Physician Accountability recommend that interviews continue to be held in a virtual format for cost, safety, and equity reasons.^6^–^10^ Residency programs may offer optional in-person visits and second looks later in the interview season, which allow applicants to see the program beyond the virtual format.^6^ In this era of virtual interviews, we consider the impact that on-site exposure, specifically away rotations, will have on applicants’ and EM (emergency medicine) residency programs’ rank list decisions.

Away rotations provide residency applicants with the opportunity to gain in-person exposure to programs beyond the virtual interview environment. While other specialties have examined the impact of visiting rotations on match outcomes, there is a paucity of such data in EM.^11^–^15^ A survey study in orthopedic surgery found that medical students applying to the specialty are now completing more away rotations than previously reported, with many matching at either a program where they completed an away rotation or at their home institution.^14^ Similarly, a recent study in plastic surgery found that completion of an away rotation was strongly associated with increased odds of receiving an interview and matching at that specific program.^15^

Whether or not the away rotation is more important now, in the virtual recruitment era, and leads to higher rotator match rates in EM remains unclear and warrants investigation. Our objective in this study was to determine the percentage of EM interns who completed an in-person rotation with the residency program in which they matched and compare this to pre-COVID-19 match rates. We hypothesized that the proportion of matched interns who rotated at their future program would increase following the transition to virtual interviews.

## METHODS

### Study Design

This was a multicenter pilot study of EM residency programs across the United States. We recruited a convenience sample of academic programs from the eight Society for Academic Emergency Medicine (SAEM) US geographic regions as defined in the SAEM Residency Directory in 2024. Students commonly use the SAEM Residency Directory when searching for away rotations in EM.^16^ We chose academic programs affiliated with a medical school to better represent the effect of a “home” rotation that is lacking at many nonacademic programs. We specifically included programs that conducted virtual-only (not hybrid or in-person) interviews post-COVID-19. A data collection tool was sent to participating sites. The tool, consisting of 12 questions, was developed by medical education faculty and a statistician at the primary author’s institution. Education faculty reviewed the survey tool for clarity and content validity before it was distributed to programs. Questions were developed in accordance with best practices in educational survey design.^17^

**Population Health Research Capsule**

What do we already know about this issue?

*Since the COVID-19 pandemic, many emergency medicine (EM) residencies have transitioned to virtual interviews, limiting applicants’ opportunities for in-person exposure to programs.*

What was the research question?

*Have EM match rates changed among in-person rotators since the shift to virtual interviews?*

What was the major finding of the study?

*Rotator match rates did not significantly differ pre/post-COVID-19 (24.7% vs 21.9%; P = .06; 95% CI*, −*6.1% to 4.0%).*

How does this improve population health?

*As applicants and programs improve their residency selection decisions and educational planning, the process will support the broader goal of improving patient care.*

We collected demographic data for each residency program including the length of each residency program, number of residents per class, whether or not the program was affiliated with a medical school, the number of years the program director (PD) had held their role, and the number of associate and assistant program directors (APD) at the program. We selected these covariates (number of postgraduate year 1 (PGY-1) EM positions, PD tenure, and number of APDs) based on prior investigations showing that PD tenure and greater faculty and resident presence on interview days may influence match rates.^3^,^18^ We also obtained the numbers of interns who previously completed an in-person rotation at the residency program in which they matched and how many matched interns were “home” versus “away” rotators. Home rotators were defined as those from the medical school affiliated with the EM residency training program. Away rotators were those from other medical schools. We also identified whether the program offered an in-person second look visit for prospective applicants and the number of those applicants who participated. Survey data was collected via Qualtrics (Qualtrics, LLC). Individual responses were de-identified.

### Study Protocol

We collected data from August to September 2024 from the participating EM residency programs.^19^ Only programs that shifted to virtual interviews in the post-COVID-19 period were included. Data were gathered for the 2017–2024 residency application years. The first 3 years (2017 to 2020) were defined as “pre-COVID” and the last three (2021–2024) as “post-COVID.” We excluded the 2020–2021 academic year given the pandemic and elimination of most away rotations during this application cycle. The data collection tool was sent via email using a Qualtrics form to residency leadership members at the participating sites who were able to extract data from their local records for the variables listed above. This study was reviewed by the institutional review board at our institution and found to be exempt from further review.

### Outcome Measures

Our primary outcome measure in this study was the proportion of EM interns who completed an in-person rotation at the residency program in which they matched, comparing pre-COVID and post-COVID years. Our secondary outcome measures included the number of matched interns who were home versus away rotators and the association between in-person second looks and intern match rates in our preliminary secondary analysis.

### Data Analysis

Data were imported from the collection tool into an Excel spreadsheet (Microsoft Corp) and situationally recoded and converted to a numeric format. There were no pre-analysis modifications in any data points, from the information submitted by survey responders. Data were then imported from the spreadsheet into Stata, version 18.5, StataCorp LLC), which we used for all analysis and plotting. Categorical data were described using proportions. For key findings (primary and secondary outcome measures) representing proportional estimates, we reported proportions with 95% CIs. We interpreted overlap of CIs as evidence against statistical significance at the *P* < .05 level. We first analyzed categorical data for the entire set of rotators (both home and away) and then repeated the analysis with focus solely on the away rotators. Paired categorical comparisons (eg, number of matched rotators in pre- and post-COVID periods) were performed using McNemar χ^2^ testing, while continuous variables were compared using a paired *t-*test.

Multivariable analysis employed logistic regression (clustered on program) to evaluate the dichotomous endpoint of whether the number of rotators rose in the post-COVID era. We prespecified three covariates as candidates for adjustment: number of PGY-1 EN positions, tenure of PD (years), and number of APDs. Logistic regression reported effect estimates as odds ratio (OR) with conservative calculation of 95% CI (based on clustering). Postestimation model evaluation assessed discrimination (*c* statistic), specification (Pregibon hat-squared), and calibration (Hosmer-Lemeshow goodness-of-fit). Additionally, autoregressive conditional heteroskedasticity (ARCH) modeling evaluated whether, within each residency program, there were any findings of sufficient volatility to invalidate combining the pre-COVID and post-COVID data.

### Subgroup Analysis

We also conducted a subgroup analysis on second look data to determine whether there was a correlation between second look visits and intern match rates. The second look results are considered preliminary, as only 8 of 11 (72.7%) programs offered a second look visit during the post-COVID era. A dichotomous variable indicating whether a program offered a second look visit was added to our logistic regression model. We then recorded the second look variable’s effect estimate (OR and its 95% CI) and statistical significance (Wald *P* value).

## RESULTS

### Descriptive Reporting of Respondent Residency Programs

Of the 13 programs invited, 11 participated in our study, yielding an 85% response rate. All respondent programs were affiliated with medical schools. Additionally, all 11 programs conducted residency interviews virtually in all the post-COVID years of the study. Seven of the eight SAEM U.S. geographic regions as designated in 2024 are represented in the data set (West excluded). Further characteristics of respondent programs are shown in the Table 1.

### Univariable Analyses

There were 384 matched PGY-1 EM residents for each of the two study eras (pre- and post-COVID). Detailed results for each of the 11 participating EM residency programs are depicted in Table 2 (see Supplemental Materials). The PGY-1 class sizes remained the same for all programs both pre- and post-COVID.

For the entire set of rotators (both home and away), 95 of 384 matched PGY-1 residents in the pre-COVID era (24.7%; 95% CI, 20.5–29.4%) had completed a rotation at the residency program in which they matched compared to 84/384 (21.9%; 95% CI, 17.8–26.3%) post-COVID. The absolute risk difference was 2.86% (95% CI, −6.13% to +4.02%) with McNemar χ^2^ *P =* .06. Based on the paired-analysis approach, for the entire set of rotators the difference in point estimates of matched PGY-1 residents (24.7% pre-COVID, 21.9% post-COVID) had a corresponding estimate of 11 more rotators pre-COVID (95% CI, −1.54 to 23.54).

When analysis focused solely on the away rotators, we found that 48 matched interns (12.5%; 95% CI, 9.4–16.2%) completed a rotation at their residency program pre-COVID compared to 47 interns post-COVID (12.2%; 95% CI, 9.1–15.9%). The absolute risk difference was 0.7% (95% CI, −10.6% to 9.1%). McNemar χ^2^ *P* value was .82. For away rotators, the estimated difference of one more rotator pre-COVID compared to post-COVID had a 95% CI of −17 to 19.

Figures 1 and 2 show results for each of the 11 programs. Figure 1 includes the total number of rotators (both home and away) who matched, while Figure 2 depicts the changes in the number of matched away rotators in pre- versus post-COVID eras. For two EM residency training programs, the proportion of away rotators was unchanged. This proportion rose for four EM programs and fell for five programs.

### Multivariate Analysis

Multivariate logistic regression analysis adjusted for three covariates (number of PGY-1 positions, tenure of PD in years, and number of APDs) while evaluating the dichotomous endpoint of whether the number of matched rotators rose in the post-COVID era. Our analyses focused on whether the proportion of all rotators (home and away) and away rotators alone rose from pre- to post-COVID. For the first logistic regression (total number of rotators), none of the three predictors were significant (all with *P* > 0.6 and effect estimates near the null). In the second logistic regression (focusing on the endpoint of whether the number of matched away rotators rose in the post-COVID era), two significant variables were identified. Both the PGY-1 EM class size (OR 3.98; 95% CI, 1.54–10.26; *P* = .004) and PD’s tenure (OR 9.78; 95% CI, 1.78–53.79; *P* = .009) were strongly and positively associated with increased likelihood of a post-COVID era rise in the number of matched away rotator students. The number of APDs was not a significant predictor (OR 1.06; 95% CI, 0.48–2.33; *P* = .89). The bivariable model’s postestimation confirmed acceptability of model discrimination (*c* = .97), specification (*hat*^2^ *P* = .96), and calibration (Hosmer-Lemeshow *P* = .81).

### Second Look Data

Of the 11 programs represented in our study, eight (72.7%; 95% CI, 39.0–94.0%) offered a second look to residency applicants for at least part of the post-COVID era. A second look was offered by one EM residency program in the 2021 match (12.5%; 95% CI, 0.3–52.7%), four programs in the 2022 match (50%; 95% CI, 15.7–84.3%), and eight programs in the 2023 match (100%; one-sided 97.5% CI, 63.1–100%). No students (0%; one-sided 97.5% CI, 0–36.9%) who completed a second look matched in the participating programs in 2021. In 2022, three EM residency programs matched a total of 14 of 18 students who completed a second look (77.8%; 95% CI, 52.4–93.6%). In 2023, six programs matched a total of 25 of 26 second look students (96.2%; 95% CI, 80.4–99.9%). The results comparing second look should be considered preliminary due to low numbers and the fact that there was variation in individual programs’ offering of second looks over the relevant period. Given these results, we found that the presence of a second look day was not associated with a program’s likelihood of matching more away rotators. The univariable absolute risk difference was 4.5% (95% CI, −2.2 to 11.2%; *P* = .34), and the multivariable odds ratio of 6.13 had a 95% CI (0.1–252) that indicated imprecision and crossed the null.

## DISCUSSION

In this pilot analysis of academic EM residency programs, we did not observe an appreciable difference in the proportion of matched interns pre- and post-COVID. Although there was a slight decrease in the proportion of matched rotators (both “home” and “away” students) who completed an in-person rotation at their residency program post-COVID compared to pre-COVID, this difference was not statistically significant. Similarly, no significant difference was found when looking at match rates of away rotators. Given the study’s relatively small and geographically limited sample, these findings should be interpreted cautiously but may provide preliminary insight into how away rotations impact match rates in the virtual recruitment era.

The correlation of in-person experiences of medical students and matching at their respective programs has been investigated in other specialties. In one study, students applying to integrated vascular surgery programs rated personal or co-applicants’ experiences as a sub-intern as one of the most important factors affecting their decision to rank and train at a specific program.^11^ Another study in otolaryngology examining the impact of the away rotation outside of students’ home geographic location both pre- and post-COVID found that the away rotation experience increased the number of interviews offered and thus increased the match rate in that targeted geographic location.^13^ Additionally, an analysis of plastic surgery programs found that there was an increased match rate for students at their home institution in the 2020–2021 cycle due to an absence of in-person away rotations.^20^ These studies suggest that in-person exposure to programs may play an impactful role on an intern’s match, which is in contrast to our findings on in-person rotations in EM. Reasons for this are unclear, but these correlations may be due to the generally smaller program size of these surgical specialties or relative competitiveness in the match. Further investigation is warranted.

The findings in our study yield several important implications for EM residency leadership, student advisors, and students applying into the specialty. The Association of American Medical Colleges (AAMC) analyzed data from an annual survey distributed to medical schools and estimated that residency interview costs for MD applicants may range from $400 to $7,000.^21^ Furthermore, a 2015 survey of fourth-year medical students found that 82% believed that rotating at a program increased their chance of matching there; the majority of away rotators spent between $1,000 and $4,000 to complete these electives.^22^ This raises a key question: Are away rotations in EM financially worth it to match at a particular academic program if match rates remain unchanged between away rotators and those who are unable to visit in person? Our data may provide valuable insight for medical students and advisors when contemplating whether it would be beneficial for students to complete multiple away rotations to match at an academic program in the specialty. It is important to note, however, that we cannot speculate on other potential benefits of an away rotation not directly correlated with matching at the program of interest such as the value of obtaining a Standardized Letter of Evaluation or advising from faculty in EM. Additionally, our findings may provide guidance for PDs as they consider whether to continue with virtual interviews or transition back to an in-person format.

We also analyzed second look data and its influence on match rates, given this is another type of in-person exposure to a program. Most EM programs included in our analysis did not track second look data for most of the study period as evidenced by our paucity of data for this variable. Because second look data were not always available or known with high reliability, these results are presented as preliminary. We believe the findings regarding second look data are best used for hypothesis-generating purposes. Further investigation is warranted to determine whether an in-person second look may replace another in-person experience such as the away rotation or in-person interview and how this may affect match rates at a particular program. While other specialties found that second looks influenced an applicant’s rank order list, finances and logistics often hindered applicant attendance.^23^,^24^ Emergency medicine applicants should weigh the financial burden of an in-person visit (away rotation or second look) with the personal benefit to make an informed decision about whether or not these are worthwhile.

## LIMITATIONS

Our study has several limitations. We included a nonrandom, convenience sample of academic EM residency programs to ensure geographic representation and for the purposes of comparing home versus away data. We also only included programs that continued virtual-only interviews postpandemic to better measure our primary aim. Our data therefore cannot be applied to nonacademic programs or programs that transitioned back to in-person interviews or to a hybrid model. It is worth noting that all applicants may not have had a uniform interview experience post-COVID-19, as some EM residency programs have transitioned to in-person or hybrid (in-person and virtual) interviews while others have remained virtual.

We recognize that this pilot study is limited by the fact that we only invited 13 academic EM programs to participate (with 11 of the 13 programs responding). Future efforts will prioritize recruitment of a larger and more diverse sample to improve generalizability and statistical power.^25^–^27^ Our sample also did not evenly represent each region, as we had more contacts with data available for all variables of interest. Specifically, the Western region, which has many EM residents, was not included and may limit extrapolation of our findings. Nevertheless, we believe these findings are valuable for applicants applying to a variety of programs in multiple geographic regions. Finally, our limited response rate on second look data should be interpreted with caution and not be applied generally. Further investigation on the impact of a second look on a larger scale is warranted.

## CONCLUSION

In this pilot study, we found no statistically significant difference in the proportion of matched interns who completed an in-person rotation in the pre- versus post-COVID-19 era. This data applies to academic EM residency programs affiliated with a medical school that conducted virtual-only interviews post-COVID-19 and in a subset of SAEM geographic regions. We must continue to evaluate the impact of virtual interviews and in-person opportunities on match rates and provide guidance on best practices to program leadership, students applying to the specialty, and student advisors in emergency medicine.

## Supplementary Information





## Figures and Tables

**Figure 1:**
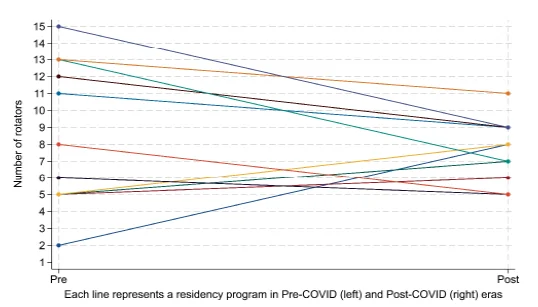
Changes in number of matched rotators for 11 emergency medicine residency programs (pre- to post-COVID). The above figure includes both home and away rotators who matched at their respective program in pre-COVID (left) and post-COVID (right) years.

**Figure 2:**
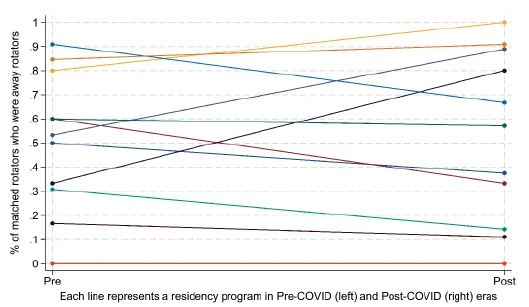
Proportion of all matched rotators who were away rotators. The figure represents changes in match rates for pre-COVID (left side of graph) and post-COVID (right) periods for away rotators alone.

**Table 1:** Characteristics of 11 responding emergency medicine residency programs in a pilot study examining match rates of rotators in pre- versus post-COVID-19 years.

Characteristic	Number (% of 11) or range	Mean (standard deviation)
SAEM US geographic region		
Northeast	2 (18.2%)	
Midwest	3 (27.3%)	
Mid-Atlantic	1 (9.1%)	
Northwest	1 (9.1%)	
Southeast/Southeastern	2 (18.2%)	
Great Plains	1 (9.1%)	
Southwest	1 (9.1%)	
Program director tenure (years)	1 to 7	3.2 (2.0)
Number of associate or assistant program directors	1 to 6	3.2 (1.5)
Duration of residency		
3 years	7 (63.6%)	
4 years	4 (36.4%)	
Total number of residents in program	18 to 63	39.0 (16.9)
Number of residents in each year		
First-year EM residents (PGY 1)	6 to 18	11.6 (3.9)
PGY 2	6 to 18	11.4 (3.9)
PGY 3	5 to 16	11.0 (3.5)
PGY 4	12 to 15	13.8 (1.5)
Affiliation with a medical school	11 (100%)	

*EM*, emergency medicine; *PGY*, postgraduate year of EM residents matched per year*; SAEM*, Society for Academic Emergency Medicine.

**Table 2:** Number of home, away, and total (both home and away) rotators matching into each EM residency training program in a study assessing rotator match rates in both pre- and post-COVID years

EM training program (Number of EM1s)	Total matching rotators (pre)	Total matching rotators (post)	Matching home rotators* (pre)	Matching home rotators* (post)	Matching away rotators* (pre)	Matching away rotators* (post)
1 (18)	2	8	1	5	1	3
2 (13)	5	6	2	4	3	2
3 (6)	5	8	2	0	3	8
4 (13)	5	5	1	1	4	4
5 (15)	6	5	4	5	2	0
6 (16)	8	7	8	3	0	4
7 (8)	11	9	1	8	10	1
8 (11)	12	9	10	1	2	8
9 (10)	13	7	9	6	4	1
10 (6)	13	9	2	3	11	6
11 (12)	15	11	7	1	8	10
Total	95	84	47	37	48	47

*EM1*, number of first-year EM residents (interns) matched per year

“Pre” refers to the pre-COVID match years (2017–2020), and “Post” refers to the post-COVID match years (2021–2024).

* Home rotators were those from the medical school affiliated with the EM residency training program. Away rotators were those from other medical schools.
