# Supplementary material for: Impact of Away Rotations on Emergency Medicine Match Rates in the Virtual Residency Recruitment Era
Source: West J Emerg Med. 2026 Oct 18;27(6):1570–7. doi: 10.5811/westjem.62267 (PMC13761836; doi:10.5811/westjem.62267)
Supplement: Supplementary file 1 [file wjem-27-1570-s001.docx]

EM Program Match Rates and Away Rotations

Start of Block: Default Question Block

We are conducting a survey to examine the impact of the emergency medicine away rotation on program match rates in the era of virtual residency recruitment. Our goal is to collect data regarding the percentage of matched EM interns who completed an in-person rotation with the residency program at which they matched and determine how this compares to pre-pandemic match rates. While we will collect demographic information about each residency program, individual survey responses will be de-identified. Survey participation is voluntary and by completing the survey, you are consenting to the use of de-identified data for research purposes. Please reach out to Jennifer Kaminsky (jkamins2@bidmc.harvard.edu) or Nicole Dubosh (ndubosh@bidmc.harvard.edu) with any questions or concerns. Thank you for your time.

Q1 Please list your residency program name here.

________________________________________________________________

Q2 What is your educational leadership role in your emergency department? (for example, program director, associate program director, assistant program director, clerkship director)

________________________________________________________________

Q3 Is your residency program a 3-year or 4-year program? Please select one of the options below.

- 3-year (1)
- 4-year (2)

Q4 How many residents are in each residency class? Please fill in number below. If you are a 3-year program, you may answer "N/A" in the PGY4 box.

PGY1 __________________________________________________

PGY2 __________________________________________________

PGY3 __________________________________________________

PGY4 __________________________________________________

Q5 How many years has your residency’s program director held this position?

________________________________________________________________

Q6 How many associate and assistant program directors does your residency program have? (Please list combined number of associate and assistant PDs).

________________________________________________________________

Q7 Is your residency program affiliated with a medical school?

- Yes (1)
- No (2)

Q8 Was your general interview season in-person or virtual for the following application cycles:

|  | Please choose one of the two options for each application cycle. | |
| --- | --- | --- |
|  | In-Person (1) | Virtual (2) |
| 2021-2022 (1) |  |  |
| 2022-2023 (2) |  |  |
| 2023-2024 (3) |  |  |

Q9 Please indicate the number of matched interns (interns who matched at your specific program) who completed an in-person rotation in your emergency department (Sub-I or another clinical rotation, i.e. pediatric emergency medicine, emergency ultrasound, etc). *Please note, we are excluding data from the 2020-2021 cycle given the pandemic changes.

- 2017-2018 (1) __________________________________________________
- 2018-2019 (2) __________________________________________________
- 2019-2020 (3) __________________________________________________
- 2021-2022 (4) __________________________________________________
- 2022-2023 (5) __________________________________________________
- 2023-2024 (6) __________________________________________________

Q10 For each of the following years, how many of those matched interns who completed an in-person rotation at your program were "home" students (i.e. your clerkship is a primary site for your medical school)?

- 2017-2018 (1) __________________________________________________
- 2018-2019 (2) __________________________________________________
- 2019-2020 (3) __________________________________________________
- 2021-2022 (4) __________________________________________________
- 2022-2023 (5) __________________________________________________
- 2023-2024 (6) __________________________________________________

Q11 Did you offer an in-person second look during the following years? Please fill in YES or NO for each application cycle.

- 2021-2022 (1) __________________________________________________
- 2022-2023 (2) __________________________________________________
- 2023-2024 (3) __________________________________________________

Display this question:

If If Did you offer an in-person second look during the following years? Please fill in YES or NO for each application cycle. Text Response Is Equal to YES

Or Or Did you offer an in-person second look during the following years? Please fill in YES or NO for each application cycle. Text Response Is Equal to YES

Or Or Did you offer an in-person second look during the following years? Please fill in YES or NO for each application cycle. Text Response Is Equal to YES

Q12 For each of the following years, please fill in the number of students who completed a second look and matched with your program.

- 2021-2022 (1) __________________________________________________
- 2022-2023 (2) __________________________________________________
- 2023-2024 (3) __________________________________________________

End of Block: Default Question Block
