# Supplementary material for: Impact of Away Rotations on Emergency Medicine Match Rates in the Virtual Residency Recruitment Era
Source: West J Emerg Med. 2026 Oct 18;27(6):1570–7. doi: 10.5811/westjem.62267 (PMC13761836; doi:10.5811/westjem.62267)
Supplement: Supplementary file 2 [file wjem-27-1570-s002.docx]

**Logistic Regression Analysis**

Data for the three match years 2017-2019 (inclusive) were defined as the “pre-COVID” period; data for the three match years 2021-2023 (inclusive) constituted the “post-COVID” period. The study’s focus on the specific before-and-after findings related to a COVID gap year (2020) prompted an approach of combining and categorizing study years. Reasonability of this combination was explored using autoregressive conditional heteroskedasticity (ARCH) modeling. ARCH evaluated for signs of year-to-year volatility, the absence of which provided support for the combining of data into two categories. Autoregressive conditional heteroskedasticity (ARCH) modeling evaluated whether, when evaluated over six years (with a gap in 2020), volatility in rotators’ numbers changed over the study period. Using the constant-only regression approach, ARCH evaluated for each residency program, the null hypothesis of no grouping of volatility into periods of high- and low-volatility. With all univariable *p* values exceeding .05, ARCH analysis was not consistent with a finding of volatility (which would have argued against combining the “pre-COVID” and “post-COVID” eras’ years).

Multivariable analysis adjusted for three covariables (EM1 positions, PD years’ tenure, and number of APDs) while evaluating the dichotomous endpoint of whether the number of matched rotators rose in the post-COVID era. Since there was particular interest in whether the number of away rotators rose, two models were generated. The first model assessed whether the number of all (i.e., total home and away) matched rotators increased, and the second model assessed whether the number of matched away rotators increased. Model-building proceeded using classical stepwise techniques, and all modeling was clustered on EMRTP (emergency medicine residency training program). The logistic regression effect metric was odds ratio (OR) with its 95% CI. Post-estimation model evaluation assessed calibration (Hosmer-Lemeshow test), discrimination (*c* statistic or area under receiver operator characteristic curve), and specification (link test).

Details on logistic regression for the main analysis are presented below. The logistic regression post-estimation evaluation suggested the model was well-calibrated (Hosmer-Lemeshow *p =* .809), well-discriminating (c = .967), and with no signs of misspecification (hat2 *p =* .956).

Table 3. Factors associated with Post-COVID era increase in number of matched rotators

| **Endpoint** | **OR (95% CI)** | ***p*** |
| --- | --- | --- |
| Increase in total number of matched rotators (home & away) |  |  |
| Total EM1 positions | 1.10 (0.75-1.62) | .615 |
| PD years in post | 1.03 (0.50-2.12) | .936 |
| APD *n* | 1.06 (0.48-2.33) | .893 |
| Increase in total number of matched away rotators |  |  |
| Total EM1 positions | 3.98 (1.54-10.26) | .004 |
| PD years in post | 9.78 (1.78-53.79) | .009 |
| APD *n* | 2.78 (0.23-33.65) | .422* |

*Abbreviations:* OR – odds ratio; CI – confidence interval; EM1 – first-year EM resident (posts to match), PD – program director, APD – Associate or Assistant PD

*The reported results for number of APDs are those obtained when forcing the variable into the model containing the other two covariates.

Details on logistic regression for the preliminary endpoint were found to be potentially unstable, with imprecise 95% CI for the effect estimate. We thus report as preliminary, the finding that presence of a second-look program was not associated with an EMRTP’s likelihood of matching more away rotators (p = .339). The precision was very low (when added to the model described in the previous section, the OR was 6.13 with 95% CI 0.15-252). We believe the findings regarding second-look data are best used for hypothesis-generating purposes.

References

1.	StataCorp. Stata time series reference manual. College Station, TX: StataCorp, 2023.

2.	Hosmer DW, Lemeshow S, Sturdivant RX. Applied logistic regression. Wiley series in probability and statistics 398. Third edition / ed. Hoboken, New Jersey: Wiley,; 2013:1 online resource.
